# Cerebral Metastasis from Breast Cancer in a Male Patient with HIV

**DOI:** 10.1155/2015/482839

**Published:** 2015-01-28

**Authors:** Guilherme Lellis Badke, Guilherme Brasileiro de Aguiar, João Miguel de Almeida Silva, Aline Lariessy Campos Paiva, Eduardo Urbano da Silva, José Carlos Esteves Veiga

**Affiliations:** Division of Neurosurgery, Department of Surgery, Faculdade de Ciências Médicas da Santa Casa de Misericórdia de São Paulo, Rua Abilio Soares 121, Apartamento 84, 04005-000 Paraíso, SP, Brazil

## Abstract

*Context*. Breast cancer (BC) in men is a rare condition, corresponding to 1% of all neoplasms in this gender. Some studies show that up to 93% of BC cases in men are advanced disease. If its occurrence constitutes an uncommon fact, the appearance of a metastasis to the central nervous system (CNS) is extremely rare. The objective of the present study is to present the case of a male patient, bearer of HIV infection, who presented with BC and later metastasis to the CNS. We also include a brief review of the literature. *Case Report*. We describe a case of a male patient, 59 years old, with HIV infection and a history of BC treated 4 years earlier, which progressed into headache and vertigo. Neuroimaging exams showed lesions suggestive of cerebral metastasis and a stereotaxic biopsy confirmed BC metastasis. *Conclusion*. Breast cancer in men with metastasis to the CNS is a rare condition and similar reports were not found in the available databases. It should be pointed out that even though rare, it should be considered among the differential diagnoses for SNC metastases in men, although HIV infection favors the appearance of some types of cancer.

## 1. Introduction

Only 1% of breast cancer (BC) cases occur in men, corresponding to 1% of all neoplasms found in the male gender [1]. Hormonal, genetic, and environmental factors explain the disease in men, but its relation with HIV remains controversial [2, 3]. Metastases at distant sites are seen in approximately 25 to 30% of the BC cases [3, 4]. The most commonly compromised sites are bones, lungs, liver, and backbone [3]. If the occurrence of BC in men is in itself an uncommon phenomenon, the appearance of metastasis of the same to the central nervous system (CNS) is extremely rare. The objective of the present study is to present the case of a male patient, bearer of HIV infection, who presented with BC and later metastasis to the CNS. We also include a brief review of the literature.

## 2. Case Report

A male patient, 59 years old, with a history of diabetes mellitus, insulin dependent, with a previous diagnosis of infection by HIV 16 years before and in regular use of antiretrovirus therapy, visited the emergency service complaining of progressive headache and vertigo. He presented with mental confusion, left appendicular ataxia, hemifacial hypoesthesia, and compromised balance with ataxy. The Karnofsky score was 30. He reported a history of cancer resection from the left breast (total mastectomy), including axillary dissection, from which the pathological and immunohistochemical investigation revealed invasive ductal carcinoma, presenting free surgical margins, without compromised axillary lymph nodes, and positive reaction to estrogen and progesterone, but HER2 (Human Epidermal growth factor Receptor-type 2) negative. He was only submitted to surgery; no complementary radiotherapy or chemotherapy was required and no signs of disseminated disease were evidenced.

**Figure 1:**
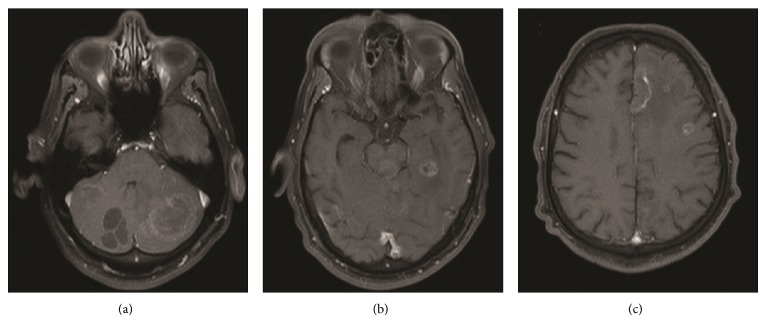
Some lesion with ring enhancement by means of contrast, sparsely in the left cerebral hemisphere, with peripheral and subcortical distribution, and also in the cerebellum.

In view of the condition, a computed tomography (CT) of the skull was requested and this revealed multiple expansive lesions, some with ring enhancement, in the left cerebral hemisphere and in the cerebellum. Magnetic resonance imaging (MRI) was requested and confirmed multiple expansive lesions (Figure 1). MRI spectroscopy analysis of midbrain and cerebellar lesion revealed an important peak in lipids with a reduced neuronal population.

**Figure 2:**
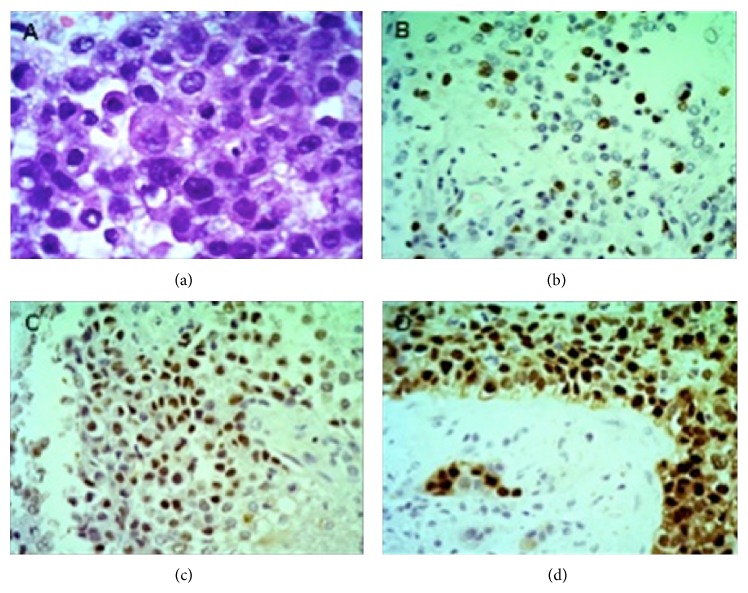
(a) Ductal carcinoma seen on hematoxylin and eosin stain with a solid pattern and a mild nuclear pleomorphism (200x). (b) Immunohistochemical stain with Ki-67 expression in 40% of the cells. (c) Immunohistochemical staining of estrogen receptor expression. (d) Immunohistochemical staining of progesterone receptor expression.

Due to the multiplicity of the lesions and the impossibility of performing a resection of the same, the patient was submitted to a stereotaxic biopsy. The result of the histopathological and immunohistochemical analyses confirmed that it was indeed a metastasis from an invasive ductal carcinoma (Figure 2). He was then referred to the oncology service, where he underwent conventional radiotherapy at the cerebral sites, with a total dose of 30 Gy in 10 fractions. Until now there were no noticed signs of another metastasis and the CNS lesions had a significant decrease disclosed by postradiotherapy MRI.

## 3. Discussion

In men, BC is a rare entity, representing less than 1% of the male gender neoplasms and corresponding to approximately 1% of all breast neoplasms diagnosed per year in the United States [3–5]. Albeit rare, in the last 25 years its incidence has shown a gradual increase. Data from the epidemiological vigilance of the National Cancer Institute point to an incidence which has surpassed 1 in 100,000 men in the 1970s to 1.2 in 100,000 men in 2000–2004. Other data show even higher incidences: from 0.86 to 1.08 in 100,000 men between 1973 and 1998, which represents an increase of 26% [3, 4, 6, 7].

It has been established that the prevalence of BC in men increases with age, reaching a plateau at 80 years of age [1], the average age at diagnosis being from 60 to 62 years [3]. Hence, BC afflicts the male population at more advanced ages than the female population by approximately 10 years [1]. The main complaints in the male patients are presence of subareolar mass (80 to 95%) [3], breast skin ulceration (45%), and nipple discharge (10%) [4]. The great majority of the cases exhibit advanced disease upon diagnosis, which can be explained, by a biologically more aggressive behavior in men [8]. Nevertheless, the main justification is the difficulty in diagnostic suspicion due to the rarity of the cases [8].

Databases show that up to 93% of the BC cases in men are of advanced disease [4], distant metastases being observed in approximately 30% of these patients upon diagnosis [3]. There are other series of cases which show different metastatic sites, among which are bones (48.78%), lungs (29.26%), liver (17.07%) [3], spine (up to 30%) [4], skin, and pleura [3]. To the best of our knowledge, there are no other cases in the literature which prove the existence of brain metastasis from BC in men.

The main BC histological subtype in men is the invasive ductal carcinoma, in 90 to 95% of the cases [3]. Other less common types are ductal carcinoma with Paget's disease and invasive lobular carcinoma [3]. The presence of compromised axillary lymph nodes has shown to be the main prognostic factor [4], with Stage T in the TNM classification upon diagnosis and onset after 65 years of age being other relevant factors [1].

The occurrence of BC in men can be favored by diverse situations, such as genetic factors, positive familial history, hormonal disturbances, occupational/environmental exposure, and genetic mutations such as BRCA [8]. It is estimated that among men with BC 10% are genetically predisposed, BRCA2 being the principal gene mutation found [8, 9]. Other reported mutations include BRCA1, P53, and CHEK 2 [9]. According to Bevier et al. [9], it was possible to detect a larger risk for women developing BC when there is a brother who has it, rather than the women whose sister is afflicted, suggesting that the genetic influence is even more important in the male gender [9].

It is known that HIV carriers can develop cancer due to the eventual immunological deficiency, the imbalance between cellular proliferation and differentiation, and disturbances in the growth factors and cytokines [10]. An extensive range of neoplasms was considered directly related to, or had an increased incidence in, people who are carriers of HIV, but BC does not seem to have an increased incidence in this population [11]. A study conducted in the United States reported that patients who were carriers of HIV possessed a relative risk of developing BC of 1.1 [12]. Despite this, only 48 cases of BC in patients who were carriers of HIV were reported up to the end of 2010 [11]. However, HIV could be related to the genesis of tumors and its aggressive behavior.

The majority of the studies performed on women did not find a difference in the incidence of BC among women with and without the HIV virus, there even being evidence that the female population carrying HIV showed lower rates of incidence of this disease, which could be explained by the immunological dysfunction of these patients [11]. There are however no studies of the same type on men. In any case, the relationship between BC and HIV remains uncertain [2].

Tumor stage and patient's general condition guide the choice of treatment for BC in men [4]. As for surgery, initial-stage disease or a locally compromised site is treated with radical mastectomy, while the metastatic disease undergoes a simple mastectomy [4]. In men, BC should be handled in the same manner as it is for women [13], radical mastectomy with axillary dissection being the surgical approach most frequently utilized [3]. Tamoxifen has proven to be a strategy capable of increasing the survival in women with BC [3]. As the hormonal receptor positivity in men is even greater than it is in women [3], a study with 57 male patients used this agent in all of the patients [4], as it is an agent capable of increasing the disease-free survival and the overall survival [14]. Radiotherapy is recommended for tumors larger than 1 cm or with more than one compromised lymph node [15], albeit some studies may recommend it for all patients with compromised lymph nodes [16]. In general, the multimodal approach, involving surgery, systemic chemotherapy, and radiotherapy, seems to be the best option for BC treatment in men [4, 17]. For patients with metastatic disease, tamoxifen can be used in combination with chemotherapy [4], which has shown good results even when the objective was palliative. In respect to the approach for central nervous system metastasis cases, up to now there is a need to broaden the concepts utilized in the treatment of women, as there are no similar cases reported in the databases consulted. The average survival is 60% for 5 years [3], 40% for 10 years [3], and approximately 20% for advanced disease [4].

Breast cancer in men with metastasis to the central nervous system is a rare condition, there being found no similar reports in the available databases. It should be pointed out that even though rare, it should be considered among the differential diagnoses for SNC metastases in men, especially if the primary site has not been ascertained. The role of HIV in the genesis of BC remains controversial, and further studies are necessary to define its influence.
